# Impact of Overnight Storage of Human Atrial Myocytes on Intracellular Calcium Homeostasis and Electrophysiological Utility

**DOI:** 10.3390/biom14111415

**Published:** 2024-11-07

**Authors:** Cristina Aceituno, David Revuelta, Verónica Jiménez-Sábado, Antonino Ginel, Cristina E. Molina, Leif Hove-Madsen

**Affiliations:** 1 Cardiac Rhythm and Contraction Group, Biomedical Research Institute of Barcelona (IIBB-CSIC), Rosselló 161, 08036 Barcelona, Spain; cristina.aceituno@iibb.csic.es; 2 Cardiac Rhythm and Contraction Group, Institut de Recerca Sant Pau, St. Antoni Mª Claret 167, 08025 Barcelona, Spain; vjimenezs@santpau.cat; 3 Institute of Experimental Cardiovascular Research, University Medical Center Hamburg Eppendorf (UKE), Martinistrasse 52, W23, 20246 Hamburg, Germany; d.revuelta@uke.de (D.R.); c.molina@uke.de (C.E.M.); 4 German Centre for Cardiovascular Research (DZHK), Partner Site Hamburg/Kiel/Lübeck, Potsdamer Strasse 58, 10785 Berlin, Germany; 5 Centro de Investigación Biomédica en Red Enfermedades Cardiovaculares (CIBERCV), Hospital de la Santa Creu i Sant Pau, St. Antoni Mª Claret 167, 08025 Barcelona, Spain; 6 Cardiac Surgery Department and IR Sant Pau, St. Antoni Mª Claret 167, 08025 Barcelona, Spain; aginel@santpau.cat

**Keywords:** human atrial myocyte, patch-clamp technique, calcium homeostasis, sarcoplasmic reticulum, calcium currents, beat-to-beat response

## Abstract

Human atrial myocytes afford an attractive experimental model to investigate mechanisms underlying electrophysiological alterations in cardiovascular disease. However, this model presents limitations, such as the availability of human atrial tissue and a variable yield of myocytes isolation. Therefore, we aimed to determine whether overnight storage can increase the time window where the electrophysiological properties of human atrial myocytes can be determined. To address this issue, human atrial myocytes isolated from patients undergoing cardiac surgery were used for patch-clamp experiments on the day of cell isolation (Day 1) and the following day (Day 2). The shape of the current–voltage (I–V) relationship for the calcium current (I_Ca_) depended on the access resistance and the cell capacitance, with large cells (>75 pF) requiring a lower access resistance (<15 MΩ) than small cells (<40 pF) to avoid distortion of the I–V curve. Importantly, overnight storage did not significantly affect (1) the I_Ca_ amplitude or properties, (2) sarcoplasmic reticulum calcium homeostasis or (3) the frequency-dependency of the beat-to-beat response. In conclusion, overnight storage of isolated human atrial myocytes at 4 °C does not affect essential features of intracellular calcium homeostasis and, therefore, affords a simple protocol to extend the experimental lifetime of human atrial myocytes.

## 1. Introduction

Human atrial myocytes afford a unique experimental model to study the impact of disease on the electrophysiological activity and contraction of isolated myocytes, and this model has, among others, been used to identify functional alterations in myocytes from patients with atrial fibrillation (AF) and the molecular mechanisms underlying the observed alterations. This includes alterations in the action potential morphology [1], the activity of ion channels [2,3,4,5,6], intracellular calcium homeostasis [7], spontaneous electrical activity [8], and the beat-to-beat response in paced myocytes [7,9]. Studies of the underlying mechanisms have, in turn, triggered research aiming to identify or test therapeutic targets specific to AF. This includes studies testing whether acute application of drugs in isolated myocytes can revert functional defects associated with AF [6,10,11,12], as well as studies where myocytes are divided into groups according to specific pharmacological treatments that donor patients have received, in order to determine the impact of the treatment on myocyte function [13]. Similarly, myocytes have been divided into groups according to clinical or genetic risk factors, concurrent disease, or other factors that could affect myocyte function [14,15,16] in order to determine the impact of these factors on cardiomyocyte function.

Some of the main limitations of using human atrial myocytes are their limited availability combined with variability in the yield of myocyte isolation and heterogeneity in myocyte quality and viability. Several approaches have been described to maximize the yield of myocyte isolation [17,18], and viral infection of human atrial myocytes has been used in different studies [19,20], supporting the notion that myocytes can maintain functional properties on the second day after isolation. However, depending on the experimental conditions, dedifferentiation of isolated myocytes kept in culture has also been reported [21,22], including changes in their electrophysiological properties [23,24]. To clarify this issue and determine if overnight storage of human atrial myocytes could yield a larger time window for electrophysiological experimentation, we focused on intracellular calcium regulatory mechanisms that mediate the excitation–contraction coupling, which is key to the regulation of both cardiac rhythm and contraction. Specifically, we compared the activity of calcium-ion channels and transporters in human atrial myocytes on the day of cell isolation and the following day.

## 2. Materials and Methods

*Myocyte isolation.* To obtain human atrial myocytes, tissue samples from the right atrial appendage of patients undergoing cardiac surgery were subjected to enzymatic digestion as previously described [7] and subjected to one or several of the experimental protocols described below. Briefly, tissue samples were cut into 1 mm^3^ cubes and incubated with magnetic stirring for 25 min in an oxygenated (continuous perfusion) enzyme solution containing calcium free Tyrode with 1 mg/mL type 4 collagenase (Worthington, Lakewood, NJ, USA), 0.25 mg/mL proteinase (sigma), 2 mg/mL fatty acid-free BSA, 5 µM Mavacamten (Selleckchem, Houston, TX, USA) plus vitamins and penicillin. Subsequently, myocytes were liberated from the tissue by gentle movements with a Pasteur pipette in calcium free Tyrode solution. The remaining tissue was subjected to additional 15 min rounds of digestion in an oxygenated enzyme solution without proteinase to maximize the yield of myocytes. All patients gave written consent to obtain the right atrial tissue samples that would otherwise have been discarded during the surgical intervention. The study protocol was approved by the Ethics Committee at Hospital de la Santa Creu i Sant Pau (AZAR-AF). Freshly isolated myocytes were stored in oxygen saturated Tyrode solution containing (in mM): 88 sucrose, 88 NaCl, 5.4 KCl, 4 NaHCO_3_, 0.3 NaH_2_PO_4_, 1.1 MgCl_2_, 0.6 CaCl_2_, 10 HEPES, 20 taurine, 10 glucose, 5 Na-pyruvate, 1 mg/mL BSA (fatty acid free), MEM vitamin solution diluted 100 times (Sigma, St Louis, MO, USA, M6895) and penicillin (pH = 7.4) on the day of cell isolation. Cells that were intended to be used on the second day rested at room temperature for up to 4 h after isolation and were then directly stored at 4 °C in a sealed vial containing Tyrode solution that was oxygenated before storage. On the following day, myocytes were acclimated for 60–90 min at room temperature before they were used for experimentation. Generally, most myocytes were used up on the second day and any remaining cells were not stored.

*Patch-clamp technique.* Only myocytes without blebs, with undamaged sarcolemma and with clear cross striations were selected for experimentation. Electrophysiological recordings (using a HEKA EPC-10 amplifier, HEKA Elektronik, Holliston, MA, USA) were performed in the perforated voltage–clamp configuration at room temperature. The intra- and extracellular solutions have been described previously [7]. The internal solution contained amphotericin B (250 µg/mL) and the extracellular solution contained 2 µM mavacamten to inhibit cell shortening, which prevents the strong cell contracture that is induced by caffeine. The steady-state L-type calcium current (I_Ca_) was measured at a pacing interval of 2 s. Currents were elicited with a 50 ms prepulse from −80 to −45 mV, followed by a 200 ms depolarization to 0 mV. The current–voltage (I–V) relationship for I_Ca_ was obtained using test potentials between −40 and +50 mV with a pacing interval of 5 s. To determine the impact of the stimulation frequency on the beat-to-beat response, myocytes were stimulated at pacing intervals of 2 s, 1.5 s, 1 s, 0.75 s, and 0.5 s. Beat-to-beat responses were classified as uniform, alternating, or irregular. The two latter responses were pooled into a group of non-uniform responses. Transient inward currents (I_TI_) activated by spontaneous calcium waves and caused by electrogenic Na–Ca exchange [25] were recorded at −80 mV in segments lasting 30 s. The caffeine-releasable sarcoplasmic reticulum (SR) calcium content was estimated by exposing the myocyte to 10 mM caffeine for 6 s. The amount of calcium released by caffeine was determined from the time-integral of the resulting transient inward current, assuming that 3 Na^+^ ions are exchanged for 1 Ca^2+^ ion by the Na–Ca exchanger (NCX). To determine the SR calcium uptake after the first caffeine pulse (Caf-1), myocytes were stimulated with 20 pulses at a pacing interval of 2 s. The time integral of the current elicited by the second caffeine pulse (Caf-2) was used as a measure of SR calcium reloading. Moreover, the change in the time constant for fast I_Ca_ inactivation (tau-1) during the 20 stimulation pulses used to reload the SR was used to determine the time course of SR reloading, assuming that tau-1 is inversely proportional to the amount of calcium released from the SR in human atrial myocytes [9].

*Data collection and statistical analysis.* Unless otherwise stated, values for quantitative variables were averaged for each patient and given as mean ± 95% confidence intervals. For normally distributed quantitative variables, the statistical significance was evaluated using *t*-test (for pairs). A two-way ANOVA was performed to analyze the effect of access resistance and voltage on the current density. For variables with clear asymmetry, statistical significance was evaluated using Wilcoxon’s rank-sum test (for pairs). Multivariate logistic regression was used to model binary variables. Analyses were performed using GraphPad Prism 9.5 or R Studio 4.2.2 software packages. Tests are indicated for each figure.

## 3. Results

**Figure 1:**
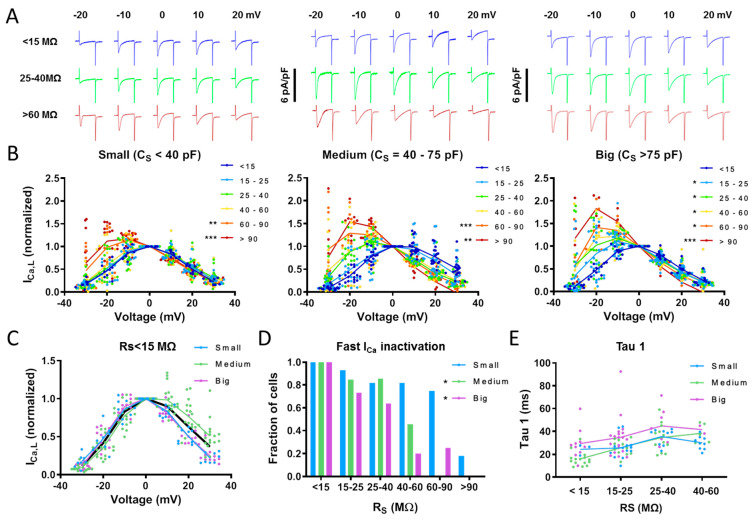
Impact of cell size and access resistance on the shape of the current–voltage relationship. (**A**) Representative I_Ca_ recordings at voltages between −20 and +20 mV at access resistances (R_s_) < 15 mΩ, between 25 and 40 MΩ and >60 MΩ. Recordings were classified according to the cell capacitance (C_S_) as small (left), medium-sized (center), or big myocytes (right). (**B**) Current–voltage relationship determined in 51 cells from 15 patients with different R_s_ given on the right. Values were normalized to the current density at 0 mV. (**C**) I–V relationship determined with R_s_ < 15 mΩ in small, medium-sized, and big myocytes. Statistical significance in panels (**B**,**C**) was determined using a two-way ANOVA followed by a Tukey’s HSD post hoc test. (**D**) Fraction of myocytes with fast I_Ca_ inactivation (tau-1) at different R_s_ given below bars. Statistical significance in panel (**D**) was determined using a multivariate logistic regression model. (**E**) Tau-1 measured at different R_s_ (given below bars) in small, medium-sized, and big myocytes. Statistical significance in panel E was determined using two-way ANOVA followed by Tukey’s HSD post hoc test. Groups significantly different from the R_s_ < 15 MΩ group are indicated with *: *p* < 0.05, **: *p* < 0.01, ***: *p* < 0.001.

Because currents were recorded using the perforated patch-clamp technique, we first assessed the relationship between cell capacitance, access resistance and the properties of the recorded currents in order to characterize the conditions that allow optimal recording of calcium currents. Figure 1A shows families of L-type calcium currents measured at different access resistances (during the perforation of the patch) for a small (<40 pF), a medium-sized (40–75 pF) and a big myocyte (>75 pF). Figure 1B summarizes the impact of the access resistance on the I–V relationship for small (<40 pF), medium-sized (40–75 pF) and big myocytes (>75 pF). Notice that the amplitude and shape of the I–V relationship could be measured correctly with an access resistance as high as 60 MΩ for small cells, whereas for cells bigger than 75 pF, the access resistance had to be lower than 15 MΩ. Figure 1C shows that when the access resistance was smaller than 15 MΩ, the shape of the I–V relationship was comparable for all cell sizes. The current density at 0 mV was −4.1 ± 0.4 pA/pF, −4.0 ± 0.4 and −3.3 ± 0.3 pA/pF for small, medium-sized and big myocytes, respectively. The fraction of cells where the time constant for fast I_Ca_ inactivation (tau-1) could be determined was significantly higher in small cells (*p* < 0.001) (Figure 1D), but the access resistance had a small effect on tau-1 (Figure 1E).

### 3.1. Impact of Overnight Storage on L-Type Calcium Current Properties

**Figure 2:**
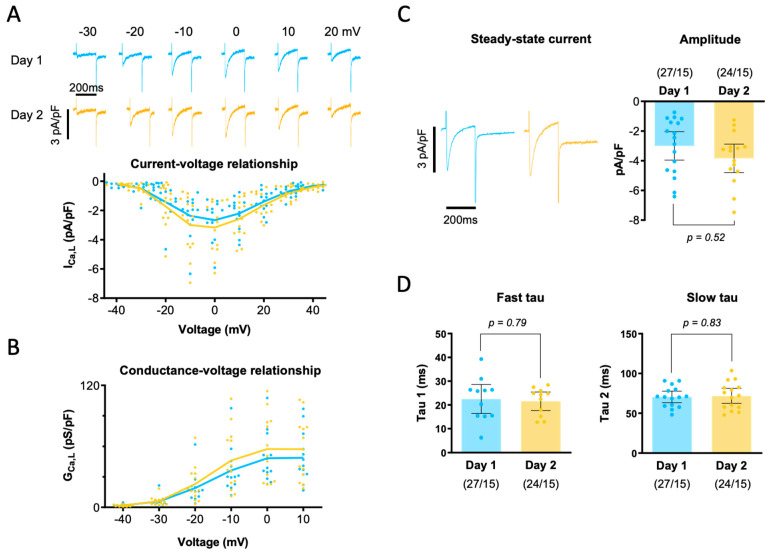
Impact of overnight storage on L-type calcium current properties. (**A**) Representative I_Ca_ traces recorded at different test potentials (given above traces) on Day 1 and Day 2 in myocytes from the same patient. The I–V relationship is shown for all myocytes. (**B**) Conductance–voltage relationship for the same myocytes. Solid lines represent average values. (**C**) Steady-state I_Ca_ recordings in myocytes paced at 0.5 Hz. The bar graph shows the I_Ca_ density for Day 1 and Day 2. (**D**) Fast and slow time constants for steady-state I_Ca_ inactivation. *p*-values given above bars were obtained using a paired *t*-test comparing data from the same patients recorded on Day 1 and Day 2.

To determine if the overnight storage of myocytes affected the I_Ca_ properties, we first compared the cell capacitance on the day of isolation (Day 1) and the following day (Day 2), and we found no statistically significant differences between the two groups (60.3 ± 8.9 pF for Day 1 vs. 64.2 ± 9.0 pF for Day 2, n = 15, *p* = 0.64). We also found that success rates were similar on Day 1 and Day 2. Thus, analysis of myocytes with an acceptable access resistance showed that the experimental protocol could be completed in 25 of 29 (86%) myocytes on Day 1, and pharmacological treatment followed by repetition of the experimental protocol could successfully be performed in 22 of the 25 myocytes (88%). On Day 2, the experimental protocol was successfully executed in 22 of 24 myocytes (92%), and pharmacological treatment plus repetition of the protocol was achieved in 19 of the 22 myocytes (86%). Subsequently, we compared the I–V relationship in the two groups. Figure 2A shows representative current recordings in the top panel, and individual data points for the I_Ca_ densities recorded at the different membrane potentials are shown below for Day 1 and Day 2, demonstrating that there were no significant differences between the two groups. This was also true for the conductance–voltage relationship (Figure 2B). Additionally, we compared the impact of overnight storage on the steady-state I_Ca_ density. As shown in Figure 2C, there were no significant differences between the two groups of myocytes paced at 0.5 Hz. Similarly, Figure 2D shows that neither the fast (tau-1) nor the slow time constant (tau-2) for I_Ca_ inactivation was different between the two groups.

### 3.2. Impact on Sarcoplasmic Reticulum Calcium Homeostasis

**Figure 3:**
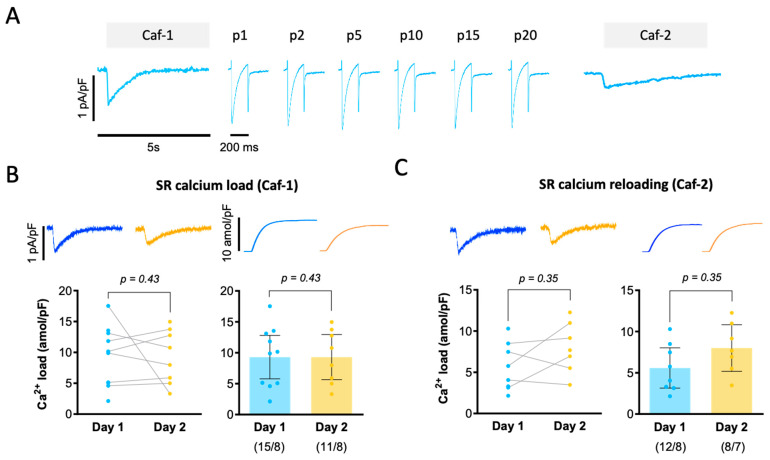
Impact of overnight storage on SR calcium loading. (**A**) Experimental protocol used to estimate SR calcium load by exposing myocytes transiently to 10 mM caffeine (Caf-1). Subsequently, 20 stimulation pulses (p1–p20) were used to reload the SR, and a second caffeine application (Caf-2) was used to determine the amount of calcium re-accumulated in the SR. Representative current traces elicited by Caf-1 (**B**) and Caf-2 (**C**) with their respective time integrals are shown on the top and mean values of the time integrals are shown below, illustrating changes on the left (paired values are connected with bars) and confidence intervals on the right. Number of cells/patients is given below bars. Statistical significance in panels (**B**,**C**) was determined using a paired *t*-test to compare data from the same patients recorded on Day 1 and Day 2.

The SR plays a key role in the activation and relaxation of contraction, and electrophysiological protocols are commonly used to estimate SR calcium uptake and storage capacity. Figure 3A shows the experimental protocol used to determine how overnight storage affects the SR calcium content and its ability to reuptake calcium after the SR calcium content has been cleared. The current traces shown in Figure 3A demonstrate that transient exposure to 10 mM caffeine (Caf), which releases calcium from the SR into the cytosol, triggers calcium extrusion from the cytosol by electrogenic Na^+^-Ca^2+^ exchange and gives rise to a transient inward current. The current elicited by the first caffeine exposure (Caf-1) reflects the steady-state SR calcium load, and the current elicited by the second caffeine exposure (Caf-2) after stimulating myocytes at 0.5 Hz for 40 s, reflects the ability of the SR to reload calcium. The time integral of the caffeine-induced current corresponds to the net charge carried by the NCX, which can be converted into moles of calcium ions carried by the exchanger. Figure 3B shows that the time integral of the first caffeine-induced NCX current is comparable on Day 1 and Day 2, suggesting that overnight storage does not alter steady-state SR calcium loading. This is also true for SR calcium re-uptake (Caf-2), shown in Figure 3C.

**Figure 4:**
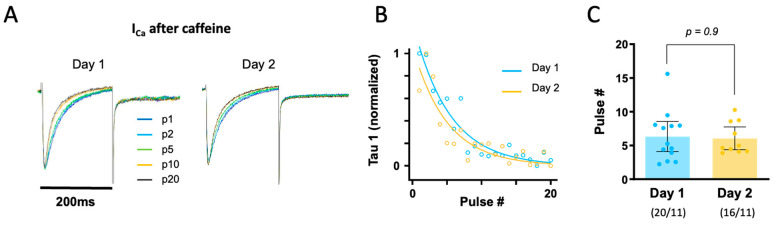
Impact of overnight storage on SR calcium re-loading. (**A**) Superimposed I_Ca_ traces elicited by stimulation pulses #1, 2, 5, 10, and 20 after Caf-1 on Day 1 and Day 2. (**B**) The time constant for fast I_Ca_ decay (tau-1) was normalized to its maximum and plotted as a function of the number of stimulation pulses used to reload the SR. Tau-1 values were fit with an exponential decaying function. (**C**) Number of pulses required to achieve half-maximal recovery of the SR calcium content on Day 1 and Day 2, assuming that it is inversely proportional to tau-1. Statistical significance in panel (**C**) was determined using a paired *t*-test to compare data from the same patients recorded on Day 1 and Day 2.

To determine the time course of calcium reuptake by the SR after the first exposure to caffeine, we measured the time constant for fast I_Ca_ inactivation (tau-1), which is inversely proportional to SR calcium loading. Figure 4A shows superimposed I_Ca_ recordings elicited by the 20 stimulation pulses used to reload the SR. Notice that the first I_Ca_ trace (when the SR is empty) has a slower inactivation than trace #20 (when the SR has been reloaded). Figure 4B compares the pulse-dependent change in tau-1 in representative myocytes from Day 1 and Day 2. Data in Figure 4B were fitted with an exponentially decaying function in order to estimate the number of stimulation pulses necessary to achieve half-maximal recovery of tau-1. Tau-1 values are shown in Figure 4C. On average, six pulses were required to achieve half-maximal recovery of tau-1, which was 24.4 ± 2.2 ms for Day 1 and 24.7 ± 2.2 ms for Day 2 (*p* = 0.9). Again, there were no significant differences between Day 1 and Day 2.

**Figure 5:**
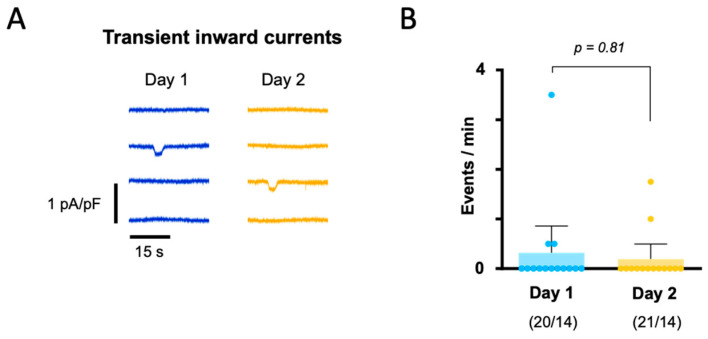
Impact of overnight storage on the incidence of transient inward currents. (**A**) Representative current traces recorded in myocytes from the same patient isolated on Day 1 (blue) and Day 2 (yellow). (**B**) Mean I_TI_ frequency recorded on Day 1 and Day 2. Error bars represent 95% confidence intervals. The *p*-value given above bars was determined using the Wilcoxon signed-rank test to compare data from the same patients recorded on Day 1 and Day 2.

To assess how overnight storage affected the incidence of spontaneous calcium release events, we measured the frequency of I_TI_s induced by the extrusion of spontaneously released calcium by the NCX. Figure 5A shows representative I_TI_ traces recorded at −80 mV, and Figure 5B shows the mean I_TI_ frequency for Day 1 and Day 2. Again, overnight storage had no significant effect.

### 3.3. Impact on the Frequency-Dependent Beat-to-Beat Response

**Figure 6:**
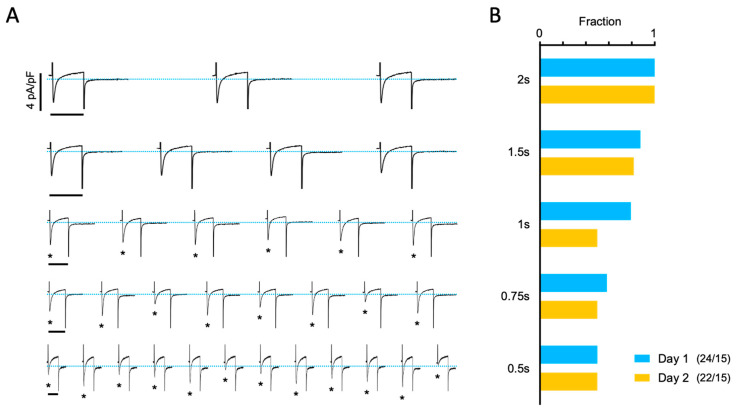
Impact of overnight storage on the beat-to-beat response. (**A**) Representative current traces recorded over a 6 s period in a myocyte paced at increasingly shorter stimulation intervals (given on the right). Horizontal scale bars indicate 200 ms for the traces. The peak I_Ca_ is indicated with asterisks at stimulation intervals of 1 s or shorter. (**B**) Fraction of 46 myocytes from 15 patients showing a uniform I_Ca_ amplitude for each stimulation frequency, recorded on Day 1 (blue bars) or Day 2 (yellow bars).

Finally, we determined the frequency-dependency of the beat-to-beat response on Day 1 and Day 2. Figure 6A shows a representative experiment illustrating the beat-to-beat response elicited when the stimulation frequency is increased from 0.2 to 2 Hz. The beat-to-beat responses were classified as uniform and non-uniform. Figure 6B shows the fractions of uniform responses on Day 1 and Day 2. A multivariate logistic regression model was used to examine the relationship between the type of beat-to-beat response (uniform vs. non-uniform) and the stimulation interval or the day that the experiments were performed. The results of this analysis showed no significant difference in the response on Day 1 and Day 2.

## 4. Discussion

### 4.1. Main Findings

The limited availability of human atrial myocytes and the heterogeneity in the quality and viability of human atrial myocytes call for the development of new protocols to improve their quality and extend their effective experimental lifetime. Here, we demonstrate that a simple overnight storage of isolated human atrial myocytes at 4 °C has no significant impact on the mechanisms that regulate intracellular calcium levels and can be measured with perforated patch-clamp technique. Furthermore, the success rate for the execution of a full experimental protocol was unaffected, doubling the effective experimental lifetime of isolated human atrial myocytes and facilitating multiple pharmacological manipulations in myocytes from the same patient. This may facilitate achieving complete dose–response curves for compounds targeting a specific mechanism, comparison of different compounds targeting the same mechanism and/or help determine crosstalk between multiple mechanisms in complex signaling pathways that regulate the activity of calcium channels or transporters.

### 4.2. Perforated Patch Recordings

The perforated configuration of the patch-clamp technique is minimally invasive compared to the ruptured patch configuration, where soluble intracellular substances are slowly dialyzed out of the myocyte into the patch pipette [26]. Indeed, modulation of calcium regulatory mechanisms by small signaling compounds, such as cyclic AMP (cAMP) that are susceptible to rundown in the ruptured patch configuration [27], has successfully been addressed with the perforated patch-clamp technique [7,10]. This includes modulation by G-protein coupled receptors [10,15], phosphodiesterases [20], as well as local subcellular regulation of calcium [7]. However, the perforated patch-clamp technique is also limited by pore formation by the compounds used to perforate the sarcolemma, which in turn can affect the quality of the electrophysiological recordings if the access resistance is too high and/or cells are too big to achieve sufficient voltage control [28]. Here, we have systematically examined how cell size, assessed from the cell capacitance and access resistance, affects the I-V relationship and inactivation of I_Ca_. Our findings show that there is an inverse relationship between the cell size and the access resistance where these I_Ca_ features can be recorded correctly. Nevertheless, the I_Ca_ features could be accurately measured in all myocyte sizes examined when the access resistance reached values smaller than 15 MΩ, which is normally attained in perforated patch experiments. However, it might be advisable to achieve an access resistance below 10 MΩ in experiments addressing the impact of compounds that stimulate the L-type calcium channel in myocytes with a cell capacitance larger than 75 pF. Theoretically, changes in the distribution of the L-type calcium current, the RyR2 or their co-distribution from Day 1 to Day 2 could affect the electrophysiological recordings reported here. However, given that we did not observe any changes on Day 2, a change in the distribution of t-tubules of calcium channels seems unlikely. Moreover, we have previously shown that their distributions remain the same in healthy and diseased human atrial myocytes [9,16].

### 4.3. Overnight Storage of Human Atrial Myocytes

The storage or culture of native human atrial myocytes beyond the day of myocyte isolation is currently used in experimentation where cells are infected with viral vectors used to genetically manipulate their properties or to introduce reporter genes [20,29,30]. However, to investigate the impact of pathophysiological conditions or pharmacological manipulations on electrophysiological features of human atrial myocytes, experimentation has preferentially been carried out on the day the myocytes are isolated. This is at least partly because the yield of human atrial myocytes tends to be low (see [18] for comparison of different protocols for isolation of myocytes from large animals), but also because storage or culture of native cardiomyocytes beyond the day of isolation has been reported to alter electrophysiological properties. Thus, it was already reported in 1996 that there is a differential regulation of voltage-activated potassium currents in cultured human atrial myocytes [31], and culture conditions also affect cell morphology and inward rectifier currents [23], as well as the T-type calcium current density [24]. Nevertheless, the present study shows that overnight storage of human atrial myocytes at 4 °C does not affect the I_Ca_ density, which is in accordance with Zorn-Pauly et al. [24], nor does it affect the time constants for steady-state I_Ca_ inactivation. The same was true for the SR calcium homeostasis, where both the caffeine-releasable SR calcium content, the time course of SR calcium refilling, and the frequency of spontaneous calcium release-induced transient inward currents were comparable on Day 1 and Day 2.

## 5. Conclusions

In conclusion, our findings demonstrate that the perforated patch-clamp technique is suitable for repetitive execution of a sequence of experimental protocols designed to address the impact of pharmacological manipulations on calcium regulatory mechanisms in human atrial myocytes provided that the access resistance is adequate, i.e., <15 MΩ for big and <40 MΩ for small myocytes. Moreover, we show that overnight storage of isolated human atrial myocytes at 4 °C affords a simple protocol for doubling the effective experimental lifetime of human atrial myocytes, which should help achieving complete dose–response curves and test compounds targeting a specific molecular mechanism underlying pathological alterations of the intracellular calcium homeostasis.

## Data Availability

The data that support the findings of this study are available from the corresponding author upon reasonable request. Some data may not be made available because of privacy or ethical restrictions.
